# Effect of hyperbaric exposure on cognitive performance: an investigation conducting numerical Stroop tasks during a simulated 440 m sea water saturation diving

**DOI:** 10.1186/s40101-024-00366-3

**Published:** 2024-10-07

**Authors:** Nozomu Kageyama, Takehito Sawamura

**Affiliations:** Undersea Medical Center, JMSDF, Tauraminatocho Mubanchi, Yokosuka, Kanagawa 237-0071 Japan

**Keywords:** Numerical Stroop tasks, Cognition, Saturation diving, Hyperbaric condition, Heliox, Stress

## Abstract

**Background:**

Saturation diving (SD) is useful and safe in deep diving for long durations. Japan Maritime Self-Defense Force (JMSDF) Undersea Medical Center (UMC) maintained safely deep 45 ATA SDHowever, cognitive performance was reportedly impaired by hyperbaric exposure in over 31 atmosphere absolute (ATA) SD. This study investigated the effects of hyperbaric exposure during 45 ATA deep SD on expert divers’ cognitive function using Stroop tasks, a useful method to examine cognitive function, especially in narrow spaces such as SD chambers.

**Methods:**

Two numerical Stroop tasks were utilized to create two magnitude comparisons of a pair of single-digit numerical and physical tasks. Both numerical Stroop tasks were examined twice, at 1 and 45 ATAs, during a simulated 440 m of sea water depth for SD. Participants were 18 male expert JMSDF SD divers (age 36.58 ± 4.89 years).

**Results:**

In the numerical task, reaction time (RT) was significantly delayed at 45 ATA compared with 1 ATA in the incongruent condition. In the physical task, RT at 45 ATA was significantly delayed under all the conditions (congruent, incongruent, and neutral). The correct rates (CR) in both numerical Stroop tasks significantly decreased at 45 ATA compared with 1 ATA in the incongruent condition.

**Conclusions:**

Our findings suggest that divers’ cognition is impaired during 45 ATA deep SD. These results emphasize the importance of monitoring cognition in deep sea SD and highlight the need to educate and train for SD. Further examination combining Stroop tasks with other analyses such as event-related potential (ERP) is expected.

## Background

In saturation diving (SD), divers stay underwater pressure until most of their tissues are saturated with breathing gas, and this allows divers to stay safe underwater in high pressure for extended durations, such as for salvage, platform construction, tunneling, and emergency submarine rescue, in both commercial companies and for the military [1–4]. It is reasonably safe and well-controlled, without causing long-term damage to divers’ health [1]. The Japan Maritime Self-Defense Force (JMSDF) Undersea Medical Center (Yokosuka, Kanagawa, Japan) has an SD program that uses a linear decompression protocol [5, 6]. Since 1977, numerous JMSDF divers have engaged in SD for emergency submarine rescue operations, salvage, and training. Using SD techniques, deep diving simulators have previously reached 450 m of sea water (MSW) at the JMSDF Undersea Medical Center and at sea [7]. The 45-atmosphere absolute (ATA) deep SD is an activity that requires heliox, a mixture of helium and oxygen. Furthermore, JMSDF SD divers have never experienced severe neurological deficits in this diving protocol [7]. Zero meter of sea water equals 1 ATA and 10 MSW equals 2 ATA. Each 10 m of sea water increases 1 ATA.

High-pressure environments impact cognitive function, causing gas narcosis or high-pressure nervous syndrome (HPNS) [8]. SD uses helium, a gas without narcotic effects. Conversely, nitrogen, a narcotic gas, is used under hyperbaric conditions. Beyond a notional threshold of 30 MSW depth (405 kPa), it can cause cognitive impairment when breathing air under 284 and 608 kPa. Furthermore, psychometric functional impairment is associated with electroencephalogram (EEG) changes in a dose-dependent manner; however, not while heliox-breathing [9]. A heliox non-saturation dive enables scuba divers’ cognitive functions to significantly increase in Stroop tasks, compared to an air dive [10]. Thus, heliox-breathing is thought to be safer than air-breathing during diving, especially for deep diving; however, helium is expensive and the cost for SD significantly increasing, limiting SD-related research.

There are several studies about the effect of hyperbaric conditions on deep SD divers’ cognition; however, this research has several notable conflicting results and inconsistencies. A study on cognitive functioning during a simulated 480 MSW heliox SD demonstrated impaired hand–eye coordination, reaction time and correct rate of mental rotation, and spatial memory. However, these impairments were limited to only four divers and showed high individual variability [11]. Other methods of studying the cognitive effects of gas mixtures at depth include critical flicker fusion frequency (CFFF) [12]. However, these studies report conflicting or paradoxical results in shallow depths (608 kPa; 50 MSW), thus, demonstrating is as an ineffective measurement tool due to low sensitivity [12]. Spatial memory, two-dimensional/three-dimensional (2D/3D) mental rotation functioning, grip strength, and hand–eye coordination abilities were also examined during a 300-m heliox SD at sea. However, the performance efficacy and mental ability were unaffected in four divers [13]. These cognitive tasks were not sensitive to impact during SD. Due to the aforementioned high cost of helium, these previous studies have a limited sample of deep SD divers, and there is a distinct lack of research on the effects of hyperbaric conditions on the cognition of deep SD divers. Our hypothesis is that robust cognitive assessment with sufficient numbers of participants could reveal how hyperbaric conditions affect deep SD divers.

**Fig. 1:**
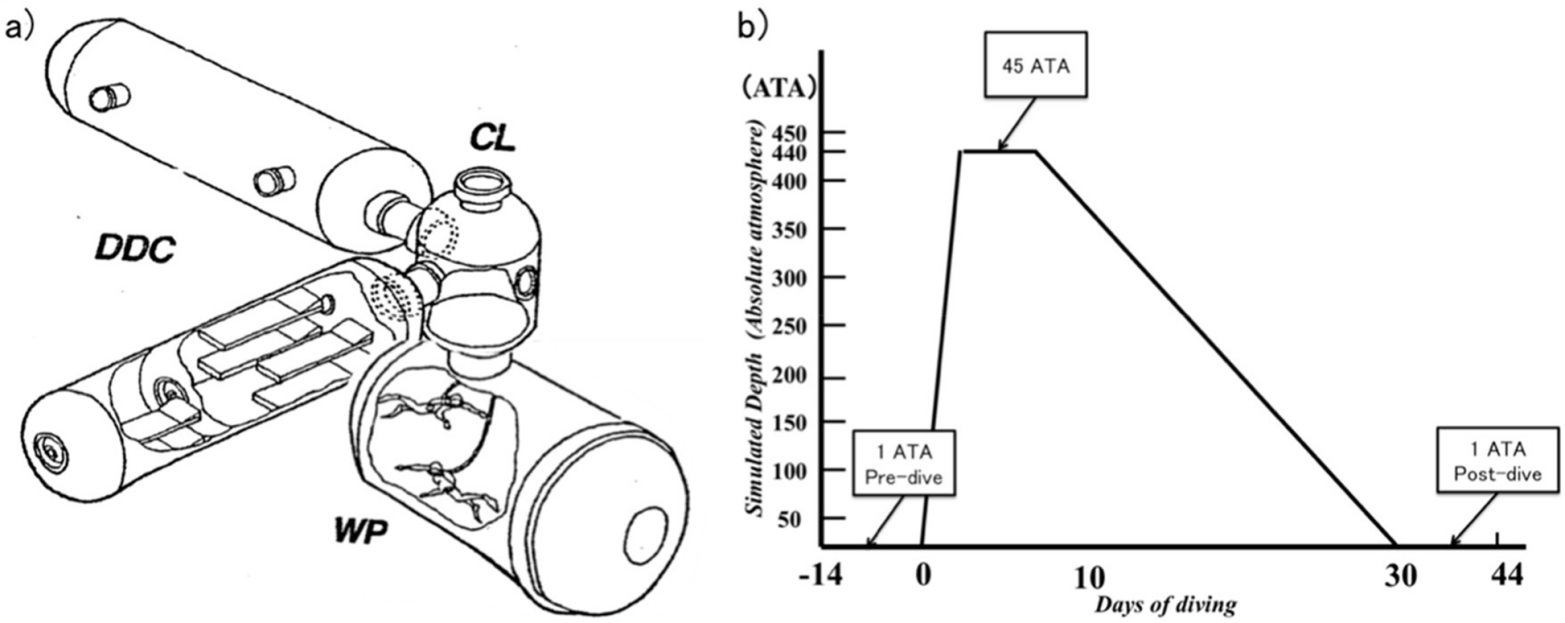
**a** The deep diving simulator (DDS) in the Undersea Medical Center, Yokosuka, Japan. DDS consists of a deck decompression chamber (DDC) for saturation diving (SD), center lock (CL), and wet pot (WP). For a basic simulated saturation dive, saturation divers are compressed in the DDC until the same pressure as working at deep sea. Second, the divers train for excursion into the simulated deep sea in WP after arriving at the pressure. They move to CL when they start training. After the excursion, they are decompressed in the DDC until the equivalent pressure of the ground (1 ATA). All experiments were conducted in the DDC. **b** SD profile and measuring points. Two type numerical Stroop tasks were administrated twice, under 45 ATA during a simulated 440 m seawater SD and under 1 ATA. Half the participants conducted the tasks pre-SD and the other examined the tasks post-SD diving

Modified versions of the Stroop task [14] are used to assess cognition with interference effects for contrariety, using different colored characters (e.g., using the word “blue” in red print), or different sized number characters (e.g., 2 7) [15–17]. Since Stroop tasks have been widely used to assess the impact of environmental stress, especially under conflict, it is expected that Stroop tasks could assess the cognition of SD divers who are under environmental stress during SD operations such as diving conditions and complex dive-related activities. Furthermore, the task can be administrated in a limited space, such as in an SD chamber (Fig. 1a), which does not permit psychometry analysis systems such as functional magnetic resonance image (fMRI), near-infrared spectroscopy (NIRS), etc., which could be easily damaged by hyperbaric conditions. Thus, we used numerical Stroop tasks including numerical tasks and physical tasks [15–17] to estimate cognitive function among JMSDF SD divers.

Stroop tasks have previously been applied to scuba-diving studies whereby participants performed the test after diving in open water or within a chamber that simulated an underwater environment [18, 19]. Stroop tasks have also been used for research in shallow diver pools (5 MSW) with anxiety estimation [20], 18 MSW dry chambers [18], and underwater (20 MSW) via a tablet [19]. However, to the best of our knowledge, no previous study has examined cognitive function via numerical Stroop tasks among deep SD divers in sufficient numbers. In the previous studies about cognition, there were too few divers and Stroop assessments in deep SD. This study design creates two-magnitude comparisons of simultaneous numerical and physical tasks in the JMSDF 45 ATA SD, which is one of the deepest heliox SD activities worldwide. Forty-five ATA is thought of as the deepest depth for heliox SD because the SD depth is limited by airway resistance. We examined cognitive function via two numerical Stroop tasks in 18 male divers using SD techniques in 45 ATA in the deep dry diving simulator at the JMSDF Undersea Medical Center. This study was also the first to estimate cognitive function with numerical Stroop tasks during deep heliox-oxygen SD. Our aim in for study was to reveal how hyperbaric conditions affect the cognition of deep SD divers using numerical Stroop tasks in a sufficient number of participants for a conclusive and in-depth analysis.

## Methods

### Participants

The participants were 18 JMSDF male divers (average age 36.58 years, standard deviation 4.89 years). Of these, 17 were right-handed and one was left-handed. All had normal vision, were highly motivated volunteers in excellent physical condition, and were trained in SD procedures (average of the total driving time: 5611.72 h, standard deviation 4287.60). All participants provided written informed consent for the experimental protocol. This study was reviewed and approved by the Ethics Committee of JMSDF Yokosuka Hospital (28–3, 29–14, and R1–08).

### Environmental conditions

All experiments were conducted in a deck decompression chamber (DDC; length 6.0 m, diameter 2.8 m) at the Undersea Medical Center, JMSDF, Yokosuka, Japan (Fig. 1a). For the hyperbaric condition, according to the SD protocol in UMC, regulated by the Ministry of Health, Labor and Welfare (MHLW), the DDC was filled with a heliox atmosphere, with a 0.42 partial pressure of oxygen (PO2) and 0.50 ATA during the dive. The partial pressure of carbon dioxide (PCO2) was kept below 0.005 ATA. Furthermore, the CO was below 40 particles per million (ppm) during the entire dive. The relative humidity in the DDC was stabilized at approximately 50–60% and the temperature was maintained between 30 and 32 ℃ (86.0–89.6 F). We should keep the room temperature warm because high-density heliox at 45ATA conducted heat away from SD divers. During SD diving in the seawater, SD divers put on SD diving suits circulated with hot water to prevent hypothermia. Figure 1b presents an outline of the diving profile. Participants were gradually pressurized to the hyperbaric condition of 45 ATA and subjected to decompression slowly over three weeks. We compared the cognitive function between 1 ATA (0 MSW) and 45 ATA (440 MSW) as the deepest depth for heliox SD.

### Tasks

**Fig. 2:**
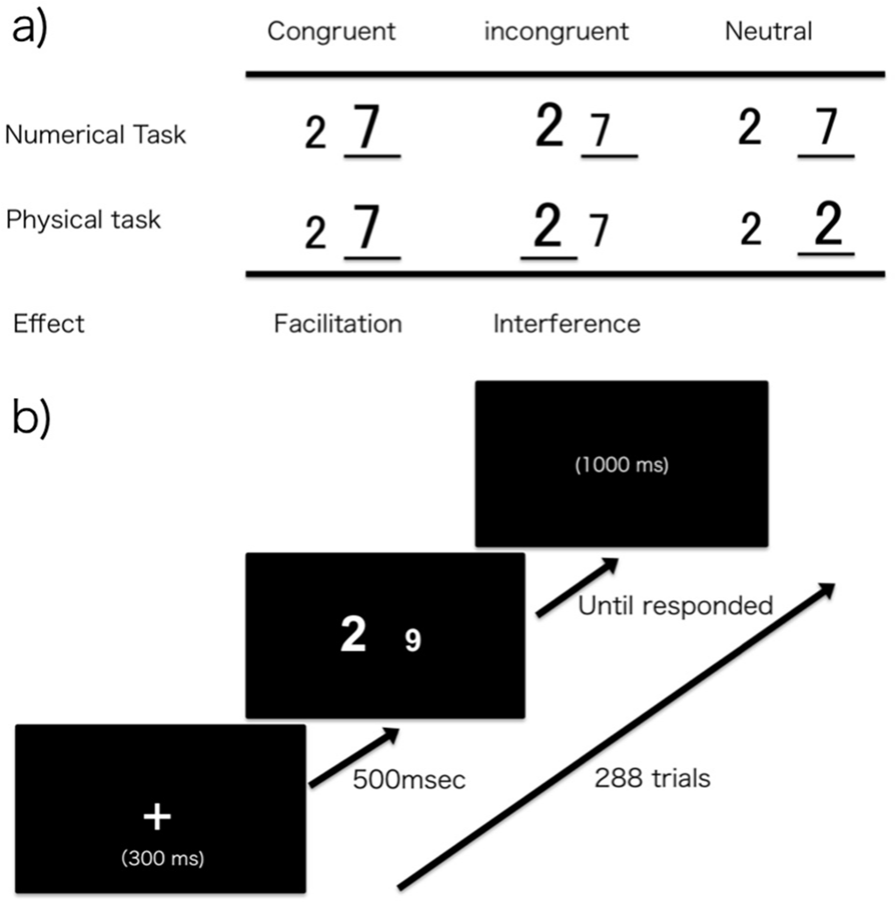
**a** Example of the stimulus pairs. The correct response is underlined. Expected effects are provided at the bottom. Facilitation and Interference are the speeding-up and slowing-down of the reaction time relative to the neutral condition, respectively. **b** General procedures of the numerical Stroop tasks. Each trial began with the fixation point shown for 300 ms. After 500 ms, a pair of stimuli were presented until the participants responded. Stimuli were followed by a pause of 1000 ms. Participants repeated 288 trials, which consisted of 192 experimental and 96 filler trials, for each experimental block (numerical and physical comparison tasks). Viewing distance was approximately 60 cm. A stimulus pair was located 7.6 and 9.5° from the top and both ends of the display, respectively

The numerical Stroop tasks aimed to capture two comparisons in the performance of numerical and physical tasks (Fig. 2a). In the numerical task, participants were exposed to a pair of digits and asked to choose the larger numeric and ignore their physical size in both the example and trials. Three stimuli were used: congruent (2 7), incongruent (2 7), and neutral (2 7); if incongruent (2 7), 7 is correct. Conversely, in the physical task, participants were presented with a pair and asked to decide the physically larger number and ignore the numerical magnitude. Three stimuli were used: congruent (2 7), incongruent (2 7), and neutral (2 2); if incongruent (2 7), 2 is correct. For neutral judgments, we used a pair of digits with the same numerical value in different sizes.

### Stimuli and apparatus

In total, six single digits were used to create the digit pairs: 1, 2, 3, 4, 6, 7, 8, 9. Four numerical distances were realized (distance 1: 1–2, 8–9, distance 3: 3–6, distance 5: 4–9, and distance 7: 2–9, 1–8). The digits were presented in Arial font with three different sizes: 67.03, 100.55, and 134.10 pixels. For both tasks, the congruent and incongruent digit pairs were always combined with a large font size difference (67.03 and 134.10 pixels). The neutral digit pairs in both tasks were displayed in Arial font with the same size (100.55 pixels) and large font-size difference (67.03 and 134.10 pixels), respectively.

The experiment was divided into two blocks based on the numerical and physical judgments. The presentation order was counterbalanced to circumvent order effects. The trial presentation was randomized within each block (congruent, incongruent, and neutral trials). Each trial began with a fixation point presented for 300 ms. After 500 ms, a pair of stimuli was presented until the participants responded, followed by a pause of 1000 ms (Fig. 2b). During the tasks, the participants held a 10-key pad in both hands. They were instructed to press “1” with the sum finger of the left hand if the left side digit was larger. Conversely, “3” was to be pressed with the sum finger of the right hand if the right-side digit was larger. In this study, the viewing distance was approximately 60 cm. A pair of digits was presented at a viewing angle of 5° from the left of the center, horizontally, while the other was displayed at the same angle away from the center, horizontally. Each experimental block (numerical and physical comparison tasks) consisted of 192 experimental and 96 filler trials, respectively. The 192 experimental trials consisted of 64 congruent, 64 incongruent, and 64 neutral trials. The 96 filler trials consisted of 32 congruent, 32 incongruent, and 32 neutral trials. Pairs of digits of semantic distance 1 and 7 were used in the experimental trials and 3 to 5 were used in the filler trials, respectively. Filler trials were used to cover the experimental trial task according to previous studies that can conserve sample size [15–17].

We controlled the timing of stimulus presentation via PsychoPy version 1.80.03 [21] and presented the pair of digits on an organic electro-luminescence display, a 17-inch Sony PVM-A170.

### Design and procedure

To achieve a 3 (congruence: congruent, incongruent, neutral) × 2 (semantic distance: 1 unit, 7 units) × 2 (environmental pressure: 1 ATA, 45 ATA) design, the following paradigm was implemented. Two numerical Stroop tasks were conducted twice, at 1 ATA (pre-dive/post-dive) and 45 ATA (Fig. 1b). To avoid the potential learning effects of the experiment, half of the participants were examined at 1 ATA before a simulated 45 ATA SD (1 ATA pre-dive) and the other half at 1 ATA after a simulated 45 ATA SD (1 ATA post-dive), counterbalancing assignment order. To avoid HPNS effects [8], we conducted the tasks two days after the final day of the paradigm simulated 45 ATA SD. We evaluated the cognitive performance under each environment via the reaction time (RT) and correct rate (CR) of the Stroop tasks.

### Statistical analysis

After confirming that the data of RT and CR were normally distributed, we conducted a repeated analysis of variance (ANOVA) for RT and CR with within-subject factors congruence (3: congruent, incongruent, neutral), semantic distance (2: 1 unit, 7 units), and environmental pressure (2: 1 ATA, 45 ATA). Post-hoc tests (Bonferroni) were conducted when significant main effects of conditions were found. SPSS version 11 was used for statistical analysis.

## Results

All 18 participants finished 45 ATA SD training without any health problems. All of them completed two numerical Stroop tasks at 1 ATA and 45 ATA. There was no difference between right-hand and left-hand when it came to pushing the 10-key pad and there were no problems regarding task presentation depending on the participants’ vision. The mean RT and CR for both numerical tasks are shown in Table 1.

**Table 1:** Mean reaction times (RT) and correct rate (CR) for different semantic distance and congruence conditions within environmental pressure in the numerical and physical tasks

Congruence	Environmental pressure	1 ATA		45 ATA	
	Semantic distance	1 unit	7 unit	1 unit	7 unit
Numerical task					
Congruent	RT (sec)	0.49 (0.11)	0.45 (0.09)	0.51 (0.07)	0.47 (0.05)
	CR	0.98 (0.02)	0.99 (0.02)	0.98 (0.03)	0.99 (0.03)
Incongruent	RT (sec)	0.59 (0.12)*	0.51 (0.08)	0.63 (0.12)*	0.55 (0.11)
	CR	0.87 (0.11)*	0.97 (0.04)	0.77 (0.12)*	0.96 (0.05)
Neutral	RT (sec)	0.52 (0.11)	0.46 (0.06)	0.54 (0.09)	0.49 (0.07)
	CR	0.97 (0.03)	0.99 (0.01)	0.96 (0.05)	0.99 (0.02)
Physical task					
Congruent	RT (sec)	0.41 (0.04)*	0.41 (0.04)	0.45 (0.05)*	0.44 (0.05)
	CR	0.99 (0.01)	0.99 (0.01)	0.99 (0.05)	0.99 (0.03)
Incongruent	RT (sec)	0.43 (0.05)*	0.45 (0.05)*	0.49 (0.06)*	0.52 (0.08)*
	CR	0.97 (0.05)*	0.93 (0.07)	0.92 (0.08)*	0.87 (0.12)
Neutral	RT (sec)	0.42 (0.04)*	0.42 (0.04)	0.45 (0.04)*	0.45 (0.04)
	CR	0.98 (0.03)	0.98 (0.03)	0.98 (0.02)	0.99 (0.01)

Standard deviations of the participant means (in parenthesis)

^*^P < .05

### Reaction time

Significant RT differences were observed between congruence and semantic distance in the numerical tasks [congruence: *F* (2,34) = 44.74, *p* ≦ 0.001, MSE = 0.0031, *η*_*p*_^*2*^ = 0.74 and semantic distance: *F* (2,34) = 47.36, *p* ≦ 0.001, MSE = 0.0036, *η*_*p*_^*2*^ = 0.72]. Post-hoc tests (Bonferroni) were conducted in congruent and environmental pressure. RTs were longer for incongruent trails than congruent and neutral trials [incongruent vs. congruent: *t* (17) = 6.97, *p* ≦ 0.001, *d* = 0.86 and incongruent vs. neutral: *t* (17) = 7.66, *p* ≦ 0.001, *d* = 0.66]. A significant interaction was observed between congruence and semantic distance.

**Fig. 3:**
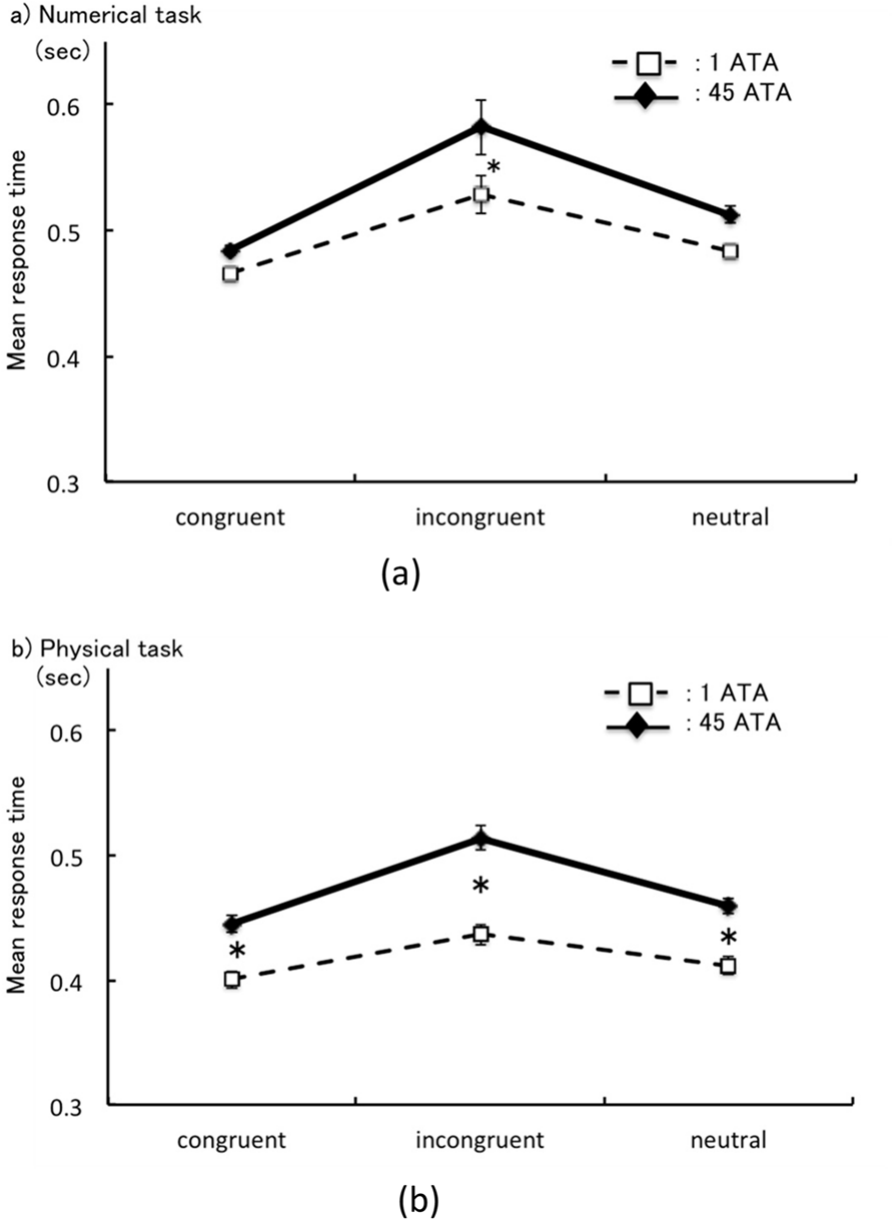
**a** Simple main effect between environmental pressure and congruency in reaction time (sec). Error bar expressed 95% CI, *p < .05. **b** Simple main effect between environmental pressure and congruency in reaction time (sec). Error bar expressed 95% CI, *p < .05

Furthermore, there was also a significant interaction between congruence and environmental pressure [*F* (2,34) = 4.61, *p* = 0.017, *η*_*p*_^*2*^ = 0.21]. RTs for incongruent at 45 ATA were longer than those at 1 ATA [incongruent at 45 ATA vs. incongruent at 1 ATA: *F* (1,17) = 6.40, *p* ≦ 0.022, *η*_*p*_^*2*^ = 0.27] (Fig. 3a). However, RTs for the other congruence conditions were not different in environmental pressure [congruent at 45 ATA vs. congruent at 1 ATA: *F* (1,17) = 0.63, *p* = 0.43, *η*_*p*_^*2*^ = 0.036; neutral at 45 ATA vs. neutral at 1 ATA: *F* (1,17) = 2.01, *p* = 0.18, *η*_*p*_^*2*^ = 0.11].

Significant RT differences were also observed between congruence and semantic distance in the physical task [congruence: *F* (2,36) = 64.64, *p* ≦ 0.001, *η*_*p*_^*2*^ = 0.79; semantic distance: *F* (1,17) = 13.40, *p* = 0.0019, *η*_*p*_^*2*^ = 0.44]. Post-hoc tests (Bonferroni) were conducted in congruent and environmental pressure. The RTs were longer for incongruent trails than those for congruent and neutral trials [incongruent vs. congruent: *t* (17) = 9.06, *p* ≦ 0.001, *d* = 0.90; incongruent vs. neutral: *t* (17) = 7.49, *p* ≦ 0.001, *d* = 0.69].

Furthermore, there was a highly significant interaction between congruence and semantic distance [*F* (2,34) = 12.75, *p* = 0.00020, *η*_*p*_^*2*^ = 0.43. In addition, RTs for all congruence levels (congruent, incongruent, and neutral) in 45 ATA were longer than those in 1 ATA [congruent at 1ATA vs. 45 ATA: *F* (1,17) = 15.97, *p* = 0.00090, *η*_*p*_^*2*^ = 0.48; incongruent at 1ATA vs. 45 ATA: *F* (1,17) = 33.81, *p* ≦ 0.001, *η*_*p*_^*2*^ = 0.66; neutral at 1ATA vs. 45 ATA: *F* (1,17) = 18.94, *p* = 0.00040, *η*_*p*_^*2*^ = 0.53] (Fig. 3b).

### Correct rate

**Fig. 4:**
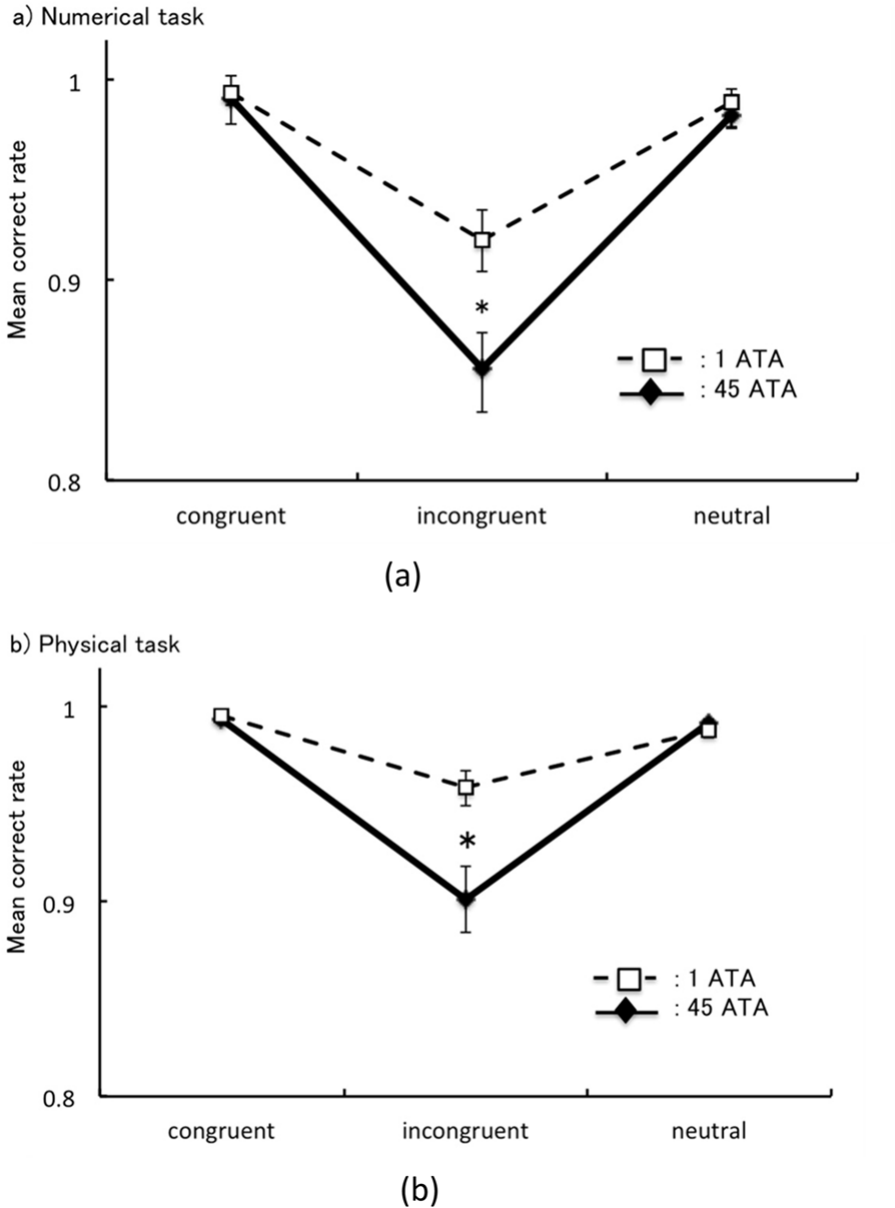
**a** Simple main effect between environmental pressure and congruency in correct rate. Error bar expressed 95% CI, *p < .05. **b** Simple main effect between environmental pressure and congruency in correct rate

There was a significant interaction between congruence and semantic distance in the numerical tasks [*F* (2,34) = 74.62, *p* ≦ 0.001, MSE = 0.0032, *η*_*p*_^*2*^ = 0.81]. Furthermore, the CR was smaller for 1 unit of distance than that for 7 units [unit 1 at incongruent vs. unit 7 at incongruent: *F* (1,17) = 91.62, *p* ≦ 0.001, MSE = 0.0065, *η*_*p*_^*2*^ = 0.84]. There was also a significant interaction between congruence and environmental pressure [*F* (2,34) = 15.75, *p* ≦ 0.0001, *η*_*p*_^*2*^ = 0.48]. In addition, the CRs for incongruent at 45 ATA were longer than those for at 1 ATA [incongruent at 45 ATA vs. incongruent at 1 ATA: *F* (1,17) = 21.61, *p* = 0.00020, *η*_*p*_^*2*^ = 0.56] (Fig. 4a). However, CRs for other congruence conditions did not differ with environmental pressure [congruent at 45 ATA vs. congruent at 1 ATA: *F* (1,17) = 0.51, *p* = 0.48, *η*_*p*_^*2*^ = 0.029; neutral at 45 ATA vs. neutral at 1 ATA: *F* (1,17) = 1.027, *p* = 0.33, *η*_*p*_^*2*^ = 0.057] (Fig. 4a).

A significant interaction was also observed between congruence and semantic distance in the physical tasks [*F* (2,34) = 4.45, MSE = 0.0037, *p* = 0.0092, *η*_*p*_^*2*^ = 0.21]. Furthermore, the CR was smaller for 1 unit of distance than for 7 units [unit 1 at incongruent vs. unit 7 at incongruent: *F* (1,17) = 5.25, MSE = 0.0055, *p* = 0.035, *η*_*p*_^*2*^ = 0.23]. There was also a significant interaction between congruence and environmental pressure [*F* (2,34) = 12.39, MSE = 0.00060, *p* = 0.0016, *η*_*p*_^*2*^ = 0.42]. The CR was smaller for incongruence at 45 ATA than that at 1 ATA [1 ATA vs. 45 ATA: *F* (1,17) = 14.48, MSE = 0.0041, *p* = 0.0028, *η*_*p*_^*2*^ = 0.46]. Conversely, no significant difference was observed between congruent and neutral at 45 ATA and those at 1 ATA [congruent: *F* (1,17) = 0.27, MSE = 0.0002, *p* = 0.61, *η*_*p*_^*2*^ = 0.016; neutral: *F* (1,17) = 0.43, MSE = 0.0005, *p* = 0.52, *η*_*p*_^*2*^ = 0.025] (Fig. 4b).

## Discussion

To the best of our knowledge, this was the first study to demonstrate that cognitive function was affected in numerical Stroop tasks in a hyperbaric condition during deep 45 ATA SD with a sufficient number of divers. Reaction times at 45 ATA were longer than those at 1 ATA for incongruent trials in the numerical task. In the physical task, the RT at 45 ATA was longer than that at 1 ATA for any congruent task. Correct rates for the incongruent task significantly decreased at 45 ATA compared with 1 ATA in both the numerical and physical tasks. These results suggest that the two numerical Stroop task performances at deep 45-ATA SD were affected by hyperbaric exposure. It suggested that the physical task might be more sensitive than the numerical task, leading to more complex cognition ignoring number content.

In SD, many factors impact cognitive functions, such as gas intake, hypothermia, HPNS, and closed space [22]. We used heliox gas, a mixture of helium and oxygen, for SD, which is the safest breathing gas without a narcotic effect [5, 10, 22, 23]. A model showing the relationship between nitrogen anesthesia and cognitive function suggests that the gas mixture did not induce cognitive dysfunction. Rather, it delayed reaction time [24]. Our finding that RT was delayed for all congruence trails in the physical tasks was consistent with this previous finding. Additionally, hypothermia during diving was an important factor to consider in cognitive impairment. We used simulated SD (dry condition), which decreased the effect of hypothermia. Möller et al. [25] reported that cognitive impairment was greater in water diving than in dry simulations. Our diving protocol was planned to prevent HPNS and our participants did not show symptoms [5]. SD divers are at risk of vascular function impairment [26], memory function with EEG change [23], another stress effect for lymphocytes [27], and hormonal changes [28]. However, our participants showed neither neurological damage nor organic changes on their medical checks, including echocardiograms and 24-h-long medical observations immediately following decompression. We found no air bubbles causing decompression embolisms in these SD divers’ vessels under echocardiogram, nor any physical or neurological disturbances during the observation periods [7]. Their impaired cognitive function was limited to the Stroop tasks and did not disturb their activity in the diving drill. The effect on Stroop tasks could not be found soon after SD without residual effects. In the JMSDF, SD divers did not show long-term irreversible damage. However, a study reported that some SD divers showed long-term chronic changes [29].

Our result suggested that hyperbaric exposure effects on cognitive function varied by the type of function. Previous studies reported that hyperbaric exposure’s impact on cognitive ability differed by the type of cognitive task [30–33]. Hyperbaric exposure affected sensory processing for perceptional information and psychomotor skills [32] and impaired executive functions and attention [31, 33]. These effects were magnified remarkably in an environmental pressure of over 31 ATA [31]. In this study, Stroop interference was used as a representative measure of executive function [34–36]. This interference has the following advantages: (1) Stroop occurred even in healthy individuals, (2) the neural basis for the generation of Stroop interference and executive function overlapped, and (3) the learning effect was small [34, 36, 37]. We found that cognitive performance in the numerical tasks deteriorated at 45 ATA compared with that at 1 ATA, suggesting that hyperbaric exposure impacted and impaired cognitive performance, particularly executive functions. While this finding was from an adequate sample, previous studies have had sample size limitations [11, 13].

According to these findings, we have modified our SD education and training to ensure that SD divers are aware of cognitive function impairment in deep SD. We emphasize the importance of physiology and psychology classes in the SD education program and focus on cognitive function change during SD diving training.

This study has several limitations. First, all participants were JMSDF male expert SD divers with enough training for SD diving. This gender-specific limitation is due to there being only one JMSDF SD female diver who has stopped her SD career. Hence, there was a trend toward a smaller hyperbaric effect on cognitive function [38]. Second, the participants might be prone to concealing their physical and psychological problems, as professional divers. We did not detect any significant changes in their daily health checks. This could lead to a scenario where their real physical and psychological changes in the numerical Stroop tasks were not accounted for. Third, we did not measure EEG or ERP in this study, which we hypothesize would reveal how hyperbaric conditions impaired cognition in the brain. We had only one previous preliminary study regarding deep SD divers with EEG and ERP at different depths [39]. Our future studies will integrate cognitive function and EEG/ERP to identify the mechanisms of hyperbaric condition effects. Fourth, we only tested 1 ATA and 45 ATA, as other depths (e.g., 31ATA, 35 ATA, and 40 ATA) do not provide enough time for cognitive assessment during linear compression/decompression safety protocols (Fig. 1b). We could not compare 45 ATA with 31 ATA/ 35 ATA for the same participants. Future studies will examine additional depth effects. Fifth, we did perform blood examinations, EEG, etc. These will be integrated into future studies. Finally, SD, especially 45-ATA, included many stress factors besides hyperbaric conditions, such as isolated solitariness, enclosed environment, difficulty in communication, and boredom. Hence, it was difficult to identify other factors that may have had confounding effects on cognition. In the future, we will examine EEGs and ERPs that are able to be used in the narrow SD spaces in order to understand what neurological mechanisms relate to impaired cognitive function. However, even with these limitations, our results provide enough information to shed light on the effect of hyperbaric conditions during 45 ATA SD on cognitive function.

## Conclusion

45-ATA SD hyperbaric conditions impact cognitive function among divers. Numerical Stroop tasks are a fruitful method to estimate cognitive function, especially in limited environments, such as SD. This study indicated that deep under-sea conditions impact divers’ cognitive performance, even though the conditions were dry, and divers were experts. However, the question still remains of how cognitive function is affected in divers’ neurological systems. Further research for safe diving should determine whether and how complex cognitive function may be affected by hyperbaric conditions, such as deep-sea SD using EEG and ERP which could be administrated in such narrow spaces as SD chamber.

## Data Availability

The datasets generated during the current study are not publicly available but are available from the corresponding author on reasonable request.

## Abbreviations

SD: Saturation diving
JMSDF: Japan Maritime Self-Defense Force
UMC: Undersea Medical Center
ATA: Atmosphere absolute
HPNS: Hyperbaric pressure neurological symptoms
RT: Reaction time
CR: Correct rate
MSW: Meter of sea water
kPa: Kilopascals
EEG: Electroencephalogram
CFFF: Critical flicker fusion frequency
2D/3D: Two-dimensional/three-dimensional
DDC: Deck decompression chamber
CL: Center lock
WT: Wet pot
PO2: Partial pressure of oxygen
PCO2: Partial pressure of carbon dioxide
ANOVA: Analysis of variance
